# The development and experience of epidemiological transition theory over four decades: a systematic review

**DOI:** 10.3402/gha.v7.23574

**Published:** 2014-05-15

**Authors:** Ailiana Santosa, Stig Wall, Edward Fottrell, Ulf Högberg, Peter Byass

**Affiliations:** 1 Division of Epidemiology and Global Health, Department of Public Health and Clinical Medicine, Umeå Centre for Global Health Research, Umeå University, Umeå, Sweden; 2 Institute for Global Health, University College London, London, UK; 3 Department of Women's and Children's Health, Uppsala University, Uppsala, Sweden; 4 MRC/Wits Rural Public Health and Health Transitions Research Unit, School of Public Health, Faculty of Health Sciences, University of the Witwatersrand, Johannesburg, South Africa

**Keywords:** epidemiological transition, demographic transition, mortality, social determinants

## Abstract

**Background:**

Epidemiological transition (ET) theory, first postulated in 1971, has developed alongside changes in population structures over time. However, understandings of mortality transitions and associated epidemiological changes remain poorly defined for public health practitioners. Here, we review the concept and development of ET theory, contextualising this in empirical evidence, which variously supports and contradicts the original theoretical propositions.

**Design:**

A Medline literature search covering publications over four decades, from 1971 to 2013, was conducted. Studies were included if they assessed human populations, were original articles, focused on mortality and health or demographic or ET and were in English. The reference lists of the selected articles were checked for additional sources.

**Results:**

We found that there were changes in emphasis in the research field over the four decades. There was an increasing tendency to study wide-ranging aspects of the determinants of mortality, including risk factors, lifestyle changes, socio-economics, and macro factors such as climate change. Research on ET has focused increasingly on low- and middle-income countries rather than industrialised countries, despite its origins in industrialised countries. Countries have experienced different levels of progress in ET in terms of time, pace, and underlying mechanisms. Elements of ET are described for many countries, but observed transitions have not always followed pathways described in the original theory.

**Conclusions:**

The classic ET theory largely neglected the critical role of social determinants, being largely a theoretical generalisation of mortality experience in some countries. This review shows increasing interest in ET all over the world but only partial concordance between established theory and empirical evidence. Empirical evidence suggests that some unconsidered aspects of social determinants contributed to deviations from classic theoretical pathways. A better-constructed, revised ET theory, with a stronger basis in evidence, is needed.

Changes in mortality patterns and increases in life expectancy, with subsequent impacts on population, have been documented in industrialised countries since the 19th century. Early studies on population change over time were overviewed from a demographic rather than epidemiological perspective (1), including Thompson's early work observing changes in fertility and mortality rates in populations (2). Landry introduced the term ‘demographic transition’ in describing secular changes in fertility and mortality in 1934, later reprinted in English (3). This idea was further developed in association with socio-economic development (4). In 1971, Omran proposed a theory of ‘epidemiological transition (ET)’, which grew out of the demographic transition model and incorporated more detailed consideration of particular diseases as causes of death. He particularly based this on mortality changes in England, Wales, Japan, and Sweden during the 19th century (5).

This ET theory, in five propositions, describes changing population patterns in terms of fertility, life expectancy, mortality, and leading causes of death (5). The first proposition states that mortality is an important aspect of population dynamics. The second proposition describes changes in disease and mortality patterns, as ‘pandemics of infectious disease are gradually shifted by degenerative and man-made diseases as the leading cause of morbidity and main cause of death’. The third proposition explains that children and young women experience the most profound impacts of ET, resulting in declining infant and maternal mortality and reduced fertility rates. The fourth proposition links long-term population changes in health and disease patterns to demographic, economic, and social determinants and mortality changes. The final proposition outlines three basic variants of ET that are functions of ‘peculiar variations in the pattern, the pace, the determinants and the consequences of population change’.

Omran proposed three stages of transition as underlying the changes in patterns of mortality and morbidity. The first stage, ‘the age of pestilence and famine’, is characterised by high and fluctuating mortality due to epidemics, famines and war, and poor living conditions. In this stage, a combination of high crude death rate, high fertility rate, and low life expectancy at birth (between 20 and 40 years) results in slow population growth. The most common causes of death are infectious and parasitic diseases, especially among children and women of child-bearing age. The second stage, ‘the age of receding pandemics’, witnesses declining mortality rates, initially high but later decreasing fertility, and life expectancy at birth increasing to around 55 years. The major driving forces in this stage of transition are sanitation improvements, control of major outbreaks of infectious diseases, and medical breakthroughs (including contraception). While infectious diseases remain as major causes of death, non-communicable diseases (NCDs) start to increase steadily. The third stage, ‘the age of degenerative and man-made disease’, is characterised by decreasing and relatively stable low mortality and increasing life expectancy at birth to over 70 years, manifesting in a population that is ageing. In this stage, NCDs dominate causes of death, with many deaths attributable to cardiac and cerebrovascular ailments, chronic lung and metabolic diseases, cancers, injuries, and stress-related disorders (5).

In 1983, Omran recognised the need to update his theory to incorporate a more extended description of the transition, as emerging analyses of transition patterns based on historical data did not fit the original model (6). Omran later acknowledged the presence of one and possibly two additional stages to his original theory of ET. He added the fourth stage as ‘the age of declining cerebrovascular mortality, ageing, lifestyle modifications and resurgent diseases’, during which life expectancy continues to increase (up to 80–85 years), and the mortality attributed to cardiovascular diseases declines and stabilises as a result of improved medical care and lifestyle modifications. Omran's fifth stage was characterised by the emergence of new diseases (HIV/AIDS, hepatitis) and re-emergence of old diseases (cholera, malaria, diphtheria, tuberculosis, plague) (7), which were already being described by others (8, 9). In his original fifth proposition, Omran proposed three basic variants of transition, but later added an additional model, similar to the classic model but starting several decades later and passing faster through the different stages of the transition (7).

## Critiques on Omran

The applicability and universality of ET theory across various places and contexts remain contentious (10–22). Criticisms of ET theory as over-simplistic peaked in the 1990s on the basis that it failed to understand the comprehensive nature and historical sequence of mortality transitions (10, 14–17, 22). A major critique of Omran's theory is the assumption that all countries will experience similar linear progression of transitions with respect to onset and speed. However, not all countries necessarily encounter ET in the same way. Omran treated entire populations as undifferentiated units; his conclusions drawn from the mortality statistics of Sweden, England, and Wales have been considered contestable; that the theory ‘fails to grasp the global nature and the historical sequence of the mortality transition as it spread’, and that it is ‘insufficiently epidemiological in that its focus was the changing causes of death rather than the changing causes of patterns of illness’ (23). Mackenbach argued that the ET theory is ambiguous because it was developed based on Western data and it is difficult to ascertain the beginning and end of the transition (20). In addition, Frenk et al. (18) and Smallman-Raynor and Phillips (19) challenged the assumptions of ET theory's unidirectional structure and continuous development, introducing the concepts of counter transition and epidemiological polarisation. It was also suggested that ET was part of a broader effort to reorient American and international health institutions towards the pervasive population control agenda of the 1960s and 1970s rather than focusing on the increasing burden of chronic disease (13).

The generalisability of ET theory has been doubted, based on the great variations in mortality trends among population subgroups (14, 21). Ruzicka and Kane examined inequalities in mortality and concluded that mortality patterns vary widely by race, sex, economic indicators, and class, resulting in substantial ‘heterogeneity within social class and within any other socio-cultural, demographic or economic category’. They criticised the ET theory for the assumption that communities will gradually progress to the point where they have virtually eliminated infectious diseases as a major health threat (15). Gaylin and Kates determined the importance of morbidity and mortality differences between population subgroups, using the HIV/AIDS pandemic as a case study, and showed important inconsistencies with the optimistic, equitable trends implied by ET theory, suggesting that the modern picture may be more complex than the original theory could predict (21).

Carolina and Gustavo examined the validity of ET theory as an effective model in the interpretation of mortality and morbidity changes, with reference to Mexico and to low- and middle-income countries (LMICs) in general. They found that the main theoretical problem in using the ET theory related to a preference for phenomenological descriptions rather than theoretical explanations of the causal patterns of death and disease and their links with the changes experienced in societies. Interpretations using the ET theory were inevitably based on scientific and social perspectives frozen at the time of its use (17). The paradox was that researchers frequently misunderstood ET theory as representing more or less the same concept as Omran's description of demographic transition, casting it as a theory about changing disease conditions progressing everywhere in a uniform and linear manner. Despite his reliance on its main concepts, Omran explicitly rejected demographic transition as a theoretical framework, postulating that ET was formulated in an attempt to provide a more comprehensive approach to the dynamics of the mortality–fertility transition. In his view, recent mortality declines in the developing world depended not on economic development but on national and international programs of health service provision and environmental control. However, this viewpoint contrasted with the thesis of McKeown et al. that broad population shifts in disease occurrence during transitions to industrialised societies, from declines in infectious disease to increases in NCDs, were due to improved nutrition and increased exposure to ‘conditions for which we are genetically ill-equipped’ (24). Preston pointed out that mortality is not associated with economic growth and the theory becomes weaker at very low levels of income (25).

In addressing the conceptual drawbacks of the theory, the term ‘health transition’ was coined to incorporate new elements into Omran's theoretical assumptions (10, 12). Health transition was described as ‘a dynamic process whereby the health and disease patterns of a society evolve in diverse ways as a response to broader demographic, socio-economic, technological, political, cultural and biological changes’, and divided into ET (changes in health patterns) and health care transition (the organised response to health conditions). Omran, however, argued that the health transition is part of the ET, not vice versa (6). The concept of ‘health transition’ was proposed as a wider framework that included not only epidemiological characteristics but also the ways in which societies responded to changing health situations as a result of cultural, social, and behavioural determinants (22, 23, 26, 27).

Clear understanding of mortality transition and its implications is still hampered due to a lack of evidence from LMICs (28). The lack of quality mortality data in many parts of the world makes it difficult to understand the generalisability of the theory globally, as well as its interpretation. Changes in disease classifications over time also limit the comparability of available data for assembling a comprehensive pattern of mortality transition (29). The theory itself received relatively little attention before the global incidence of NCDs increased in the 1990s. An overview of how the ET theory has been applied since its conception, and the identification of gaps where it fails, are warranted.

## Objectives

This paper intends to synthesise published evidence on mortality transition, and, if possible, assess how ET theory has been applied in understanding the transition in specific contexts. More specifically, this paper aims to answer the following questions: 1) What evidence on mortality transition is available, who are the beneficiaries during the transitions, and what are the social–economic determinants that coexist with the ET?; 2) What existing evidence is available to illustrate changing patterns of causes of death?; and 3) What deviations from the classic ET theory have been observed, and do these reveal emerging patterns?

## Methods

We conducted a systematic review in PubMed using the keywords ‘epidemiological transition (s) or epidemiologic transition(s) or demographic transition(s) or health transition(s)’ and ‘mortality’. We selected these keywords to cover a wide range of transitions, including health and demographic transitions that are related to ET. We included only articles on human research, and published in English between 1 January 1971 and 31 December 2013.

**Fig. 1:**
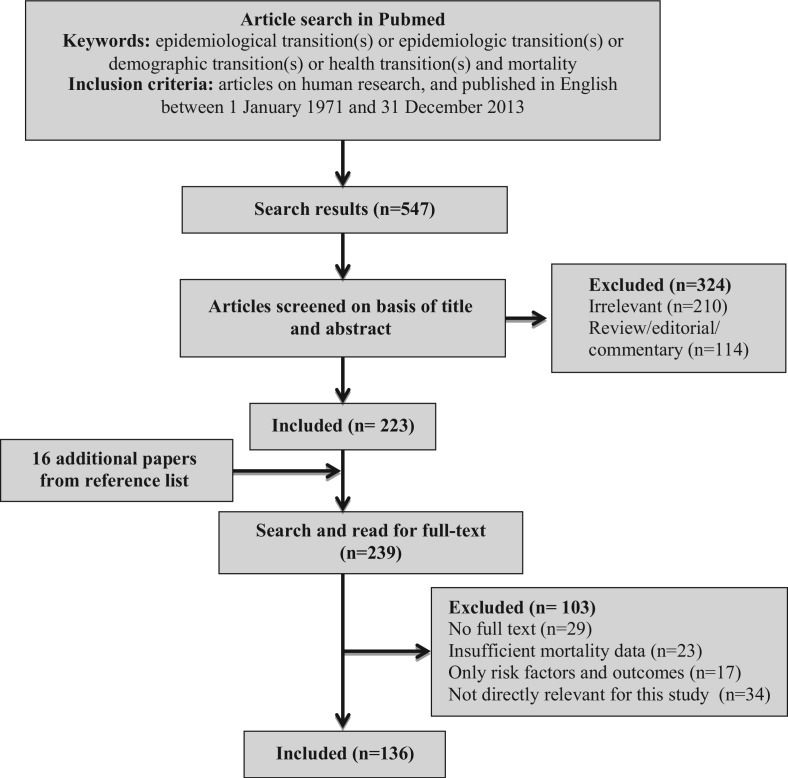
Flow diagram of the literature search process.

We obtained 547 articles, which were later screened by reading their titles and abstracts. From this step, a total of 324 articles were excluded (210 irrelevant papers and 114 reviews/commentaries/editorials). Review papers including Omran's and Caldwell's conceptual papers were excluded but used to provide framework in the ‘Discussion section’. The full texts of remaining 223 articles were searched and read through. From this step, we obtained 16 additional articles, not found in the original search, from the reference lists of relevant studies and review articles. We could not obtain full text for 29 papers. Therefore, only a total of 210 articles were included for full text review in the second step. All citations were saved in the PubMed database and imported into the EndNote X6 database. All full-text articles were reviewed by two of the authors for inclusion in the study. Uncertainties over study inclusion were discussed between the researchers and resolved through consensus. Another 74 papers were further excluded after reading the full text, mainly because of insufficient mortality data (*n*=23), observational studies assessing risk factors and outcomes in a defined population (*n*=17), and papers not directly relevant to this study (*n*=34). A final 136 articles were included in the review. Details of this literature search are summarised in Fig. 1.

## Results

Of the 136 articles, 112 articles were performed in single countries, and the other 24 articles were conducted in multiple countries in which some of multi-countries studies used the Global Burden of Disease models. Seventy-five out of the 136 articles (55%) used individual-level data, and the remaining 45% used ecological data. Fifty papers (37%) used historical data before Omran's theory was postulated in 1970, and the remaining 63% focused more on contemporary society after 1970. Seventy-nine of the articles (58%) reported time trends in outcome indicators, while the remaining 42% reported cross-sectional observations.

The main outcome indicator used in most studies was mortality rate (either total, sex specific, age specific, and/or cause specific), which was used in 117 articles (87%). The remaining 13% articles used absolute number or proportion of deaths (9%), DALY (3%), life expectancy and standardised mortality ratio (1%). On disease outcomes reported, 49% of the papers examined both all and cause-specific deaths, 27% only focused on all-cause deaths, and 24% reported cause-specific deaths.

With regard to the research questions, 50 papers (37%) assessed trends in outcome indicators, with 37 additional papers (27%) analysing both the trends and their determinants. Twenty-three papers (17%) reported the prevalence of the outcome indicators, and 21 additional papers (15%) reported both the prevalence and their determinants. Five papers (4%) reported both the prevalence and the trends of the outcome indicators. Twenty-nine papers (21%) reported results from studies in rural areas, 1% in urban areas, 16 papers (12%) in both urban and rural areas, and 90 papers (66%) did not characterise the study area. A total of 112 papers (82%) reported results from studies in general populations, 16 papers (12%) on children under 15 years, and eight papers (6%) on adults aged 15 years and above (Table 1).

**Table 1:** Regions and countries from where the papers included in this review originated (n=136 papers)

	Papers
Africa	Ethiopia (30, 31), Ghana (32), Malawi (33), Mauritius (34), Morocco (35), Seychelles (36, 37), South Africa (38–44), Sub-Saharan Africa (45), Tanzania (46)
America – North	Canada (47–52), Cuba (53, 54), Costa Rica (55), Haiti (56), Jamaica (57), Mexico (17, 58–63), Trinidad and Tobago (64, 65), USA (66–68)
America – South	Brazil (69–74), Chile (75), Peru (76, 77), Colombia (78)
Asia	China (79–83), Hong Kong (84), India (25, 85–89), Israel (90), Japan (91, 92), Korea (93–95), Palestine (96), Russian (97), Singapore (98), Sri Lanka (99), Thailand (100, 101), Turkey (102), Vietnam (103, 104)
Europe	Bolivia (105), Bulgaria (106), Czech Republic (107), France (108), Hungary (109), Lithuania (110, 111), The Netherlands (112, 113), Spain (114–119), Sweden (120–123), United Kingdom (124, 125)
Pacific	Australia (126, 127), Fiji (128), French Polynesia (129, 130), Nauru (131, 132), New Zealand (133, 134)
Multi-countries	WHO databases, mainly from World Health Statistics and Global Burden of Disease database (135–150), national data (151–156), Human mortality database (157–159), Demographic Health Surveillance System in INDEPTH Network (160, 161), other databases (147, 162–164)

## Discussion

Omran's theoretical concept of ET has broadly been used in public health because it offers explanations for changing cause-of-death patterns corresponding to different stages of transition. Most studies reviewed in this paper described the theory in relation to specific situations in particular countries, rather than considering its generalisability. Our review attempts to overview published evidence over four decades on mortality transition in relation to the ET theory. We structure the discussion in several sections to discuss how the empirical research articles illustrate the overall and cause-specific mortality transitions, and whether deviation from the ET theory and emerging patterns have been observed. A comprehensive exploration of health and ET in each country included in this review, as well as a thorough review of historical demographic research that led to Omran's theory, is beyond the scope of this paper.

### What evidence on overall mortality transition exists?

Many studies have confirmed Omran's first proposition on ‘mortality as a fundamental factor in population dynamics’. In many countries, mortality in the population decreased significantly in the past century, alongside decreasing fertility, increasing life expectancy, and a growing number of older population. In this section, we review papers, which discuss how transition of mortality has occurred and how it influenced the population structure over a period of time.

Mortality decline has been observed with different speeds of transition in the population among all age groups (17, 32, 34, 53, 55, 61, 65, 67, 75, 86, 91, 98, 106, 114, 116, 123, 124, 145) and across specific age groups, including neonates and infants (50, 99, 117), children (35, 56), adolescents (142), and adults (73, 85, 127). In some countries – such as Spain, Japan, Mexico, and Sri Lanka – good quality vital registration allows researchers to study mortality transition over long time periods. The mortality rate in Spain increased during the 19th century and peaked at the end of the century, after which the rate decreased at a rate of 2.3 deaths per 1,000 population per decade during the 20th century (114, 116). During the 20th century, many countries experienced quite similar reductions in death rates with a reduction between 2 and 3 deaths per 1,000 population in a decade, such as in Japan (91) and Mexico (17). These data illustrate how the speed of mortality transition has become faster over time.

Decreasing fertility and birth rates led to an increase in life expectancy and a change in the population structures (35, 75, 114). While the mortality rate in Spain started decreasing by the end of the 19th century, the birth rate did not start declining until 1930. Since then, it has decreased by two-thirds and reached the level of 9.75 per 1,000 population by the end of the 1980s. The proportion of population dying at 70 years or older changed from 25% in the beginning of the 19th century to 74% by the end of the 20th century (114). Chile experienced a faster transition, with the proportion of deaths among older people over 65 years doubling from 35 to 60% during a 22-year period (1970–1992) (75). In Monaco, the fertility rate halved within a 30-year period (1980–2011), with 29% of the population being children under 15 years and 7% older people aged 65 +^35^.

In contrast to the general pattern of decreasing mortality during the 20th century, populations in several settings experienced stagnation of mortality reductions, such as Eastern European countries (147), and even reversal of mortality, such as in Nauru (131), Philadelphia in the USA (66), Russia (152), South Africa (38–40), Tanzania (46), and Thailand (101). A high rate of tuberculosis mortality among the adult population, which slowed down the increase in life expectancy, was observed in Philadelphia during the beginning of the 20th century (66). In Nauru, the age-standardised mortality rate among adult men doubled during 1960–1981, mainly due to the high burden of chronic NCDs (131). In rural South Africa, Kahn et al. demonstrated significant increases in mortality for men and women, mainly due to the HIV epidemic, during the last decade of the 20th century, with life expectancy decreasing rapidly by 12 years in females and 14 years in males in a 10-year period (38). The overall mortality rate increased by 87% from 1992 to 2005 (39). In Thailand, the generalised HIV epidemic since the 1990s has led to an increase in male mortality rate and a decreased life expectancy by 4 years during 1990–2000 (101).

Some studies have been conducted in indigenous populations in Bolivia (105), Canada (50, 52), Mexico (61), New Zealand (133), and Sweden (120, 121). Indigenous populations tend to have higher birth rates, mortality rates, and lower life expectancy than the general population, as has been observed among sub-Arctic Indians in Canada (50) and the aboriginal Maori population in New Zealand (133). The Zapotec-speaking genetic isolate in the Valley of Oaxaca, southern Mexico, experienced mortality crises during 1930–1950 with the measles epidemic, resulting in deaths exceeding births. After 1955, birth and death rates diverged in a pattern typical of rapid population growth in early Stage II of the demographic transition (61). Recent evidence has shown that health among indigenous populations has improved over time, as has been observed among the Sami population in Sweden by the end of 19th century (120), and among the Tsimane Amerindian population in Bolivia during the second half of the 20th century (105). In Sweden, the gaps in infant mortality rate (IMR) between the Sami and non-Sami populations became narrower over time. Despite these improvements, health inequality persisted between the south Sami and the north Sami (121).

#### Evidence of transition among children and young women

In his third proposition, Omran stated that ‘During the epidemiologic transition the most profound changes in health and disease patterns obtain among children and young women’. Evidence from many countries in this review supports this claim (17, 32, 35, 53, 56, 58, 64, 66, 67, 72, 75, 80, 86, 95, 102, 107, 114, 116, 124, 128, 142, 146, 158). For example, the IMR and perinatal mortality rate decreased steadily in Chile during 1970–1992, while the neonatal mortality rate has been stable since the 1970s (75). During 1992–2011, the mortality rate among children in Morocco decreased by more than 40% over a 20-year period (a decrease of 44% in neonatal mortality rate, 54% in IMR, 64% in under-five mortality rate, and 66% in postnatal mortality rate) (35). Sastry reported significant decrease in under-five mortality from 117 per 1,000 live births in 1970 to less than 50 by 1991 in Brazil (72).

In some countries, however, sustainable health improvements among children and young women were not observed during the transition. Infant mortality among Tsimane population in Bolivia did not decrease significantly concomitantly with large reductions in adult and old age mortality during 1950–2000 (105). Child mortality in Nauru fluctuated and increased over time from 6 to 10.8 per 1,000 population during 1960–2007 (131). Unlike other countries in South East Asia that experienced a decline in infant mortality during 1960–1980, Thailand still struggled with high infant mortality of 40–45 per 1,000 live births in the 1980s (154). Kahn et al. showed that children (0–4 years) and young adults (20–49 years) in rural South Africa experienced rapid increases (two and five times, respectively) in mortality rates over a 10-year period due to the HIV/AIDS epidemic (38, 40). In South Africa, the HIV/AIDS epidemic led to the disease becoming the leading cause of death among infants and young adults by 2000. Women suffered more from HIV/AIDS and chronic diseases than men (42).

### What evidence is available to illustrate changing patterns of causes of death?

In his second proposition, Omran postulated ‘During the transition, a long-term shift occurs in mortality and disease patterns’. Omran suggested a shift in the causes of death from predominating infectious diseases to NCDs. Therefore, we tried to identify how countries included in this review stood in terms of ET stages over time and later discuss specific transitions that did not follow the patterns proposed by Omran.

Table 2 shows a summary of countries at various ET stages over time. Industrialised countries generally started ET earlier but proceeded slowly. Western European countries such as Sweden, United Kingdom, and France took most of the 19th century to shift from Stage 2 to Stage 3 and then a further half-century from Stage 3 to Stage 4 (108, 122, 124, 165). Cuba, USA, and Australia entered Stage 2 later than many European countries (53, 54, 66, 67, 126) but reached Stage 3 almost at the same time. The Netherlands (113) faced Stage 1, ‘the age of famine and pestilence’, when USA and Australia were encountering Stage 2. Hungary moved rapidly from Stage 2 to Stage 3 at the end of the 20th century, after the rapid political changes, and is now in Stage 4 of ET with declining cardiovascular mortality and increasing life expectancy (109). The same stage, with declining cardiovascular mortality, was observed in Canada during 1978–1996 (51). In the former Soviet Union, ET did not progress homogeneously across different geographical regions in the country. The male population in the northern part experienced higher mortality due to chronic diseases, such as cardiovascular diseases, injuries, and lung cancer (166).

**Table 2:** Timeline of selected countries reported to be at various stages of epidemiological transition

	Stage 1	Stage 2	Stage 3	Stage 4
1870–1874	United Kingdom (153, 157), Sweden (153, 157), Finland (153, 157), Iceland (153, 157), USA (66), Germany (167)			
1875–1879	The Netherlands (113)			
1880–1884				
1885–1889				
1890–1894				
1895–1899				
1900–1904	Spain (114, 116, 117)			
1905–1909				
1910–1914		United Kingdom (124, 153, 157), Sweden (153, 157), Finland (153, 157), Iceland (153, 157), The Netherlands (113)		
1915–1919				
1920–1924	Mexico (17)	Spain (114, 168)		
1925–1929				
1930–1934		Germany (167)		
1935–1939	Japan (91)			
1940–1944				
1945–1949		Costa Rica (55)		
1950–1954	Hong Kong (154), Trinidad and Tobago (64, 65)	Japan (91, 158), Ghana (32), Canada (47, 169)	The Netherlands (113), United Kingdom (124, 153, 157), Sweden (153, 157), Iceland (153, 157)	
1955–1959		China (158)		
1960–1964			Cuba (140), Puerto Rica (140)	
1965–1969		Hong Kong (154), Singapore (154)	Spain (114, 116, 117), Costa Rica (55)	
1970–1974	Peru (77)	Trinidad and Tobago (64, 65)	Japan (158), Mauritius (34), Canada (169)	Netherland (113)
1975–1979		Korea (95)		
1980–1984				
1985–1989		Mexico (59, 140)	Hong Kong (154) Malaysia (154)	Costa Rica (55), Canada (47), Spain (168)
1990–1994		Peru (77)	Seychelles (37), Ghana (32), Cuba (54)	Japan (158), France (108), Australia (126, 127), Trinidad and Tobago (65)
1995–1999			Mexico (17), Korea (93)	China (158)
2000–2004			India (85, 86), Thailand (100, 101)	Mexico (60), Peru (76), Hungary (109), Brazil (69, 73, 118)
2005–2009				Vietnam (104), Mexico (170)

Spain entered Stage 1 at the beginning of the 19th century (116, 117). The number of deaths have fluctuated and have slightly increased since the beginning of the 19th century in Spain, with several severe mortality crises including the cholera epidemic in 1855, the influenza pandemic in 1918, and the nutritional deficiency problem during the Spanish Civil War in 1941. At the beginning of the 20th century, circulatory mortality replaced infectious diseases as the leading cause of death (114). By the end of the century, circulatory disease was responsible for 75% of total deaths and less than 5% deaths were related to infectious disease, a characteristic of Stage 3 (114). The fourth stage, which is characterised by declining circulatory disease, increasing degenerative diseases, such as malignant neoplasms and metabolic disease, and a stable, low proportion of infectious disease, was observed in Spain at the beginning of the 21st century (119).

Up to the mid-20th century, average life expectancies in LMICs were much lower than those of industrialised countries, largely driven down by premature infectious disease mortality. After World War II, most countries progressed faster and followed a general trend of converging and increasing life expectancy. Most LMICs entered Stage 2 later than high-income countries (in the second half of the 20th century), but they subsequently moved on to the next stages of ET more rapidly than that of the high-income countries. This faster transition was observed in Brazil (70, 74), Chile (75), China (79, 80, 155), Japan (91), Korea (93), Mexico (60), Peru (76, 77, 171), and Vietnam (104), perhaps because of greater economic growth and improvements in health. By the end of the 20th century, the proportion of ailments related to circulatory disease had increased to 75% in Chile (75). Diabetes mortality in Mexico increased slightly during 1980–2000 (62). By 2004, Mexico had progressed further in the ET, with NCD and injury as a major burden of disease [75% of all deaths in 2004 were due to NCDs, mainly due to ischaemic heart diseases (13%), diabetes mellitus (10%), cerebrovascular diseases (6%), liver cirrhosis (6%), and road traffic accidents (4%)] (60).

A variant of the non-Western transition model has been formulated as the ‘protracted polarised transition model’, in order to account for the process of epidemiological polarisation. Frenk et al. conceptualised this extended variant of the ET theory based on Latin American data. Latin America had experienced a resurgence of malaria and dengue fever, along with a burden of infectious diseases and NCDs across regions and social classes, with widening health gaps across regions and social classes due to an unequal distribution of health interventions. The epidemiological polarisation element is characterised by the overlap of eras, a persistence of infectious diseases combined with the emergence of NCDs and new epidemic diseases, capturing also increased geographical and social health inequalities. They also showed several main features of the protracted–polarised model; that the period of mortality decline is short in contrast with the classical model; the onset of mortality decline does not begin before the 20th century; infectious diseases are not yet completely controlled, with consequent overlaps of stages and unequal distributions of wealth and health; and re-emergence of old diseases that did not feature in the original ET theory (18).

Evidence from some countries confirmed the protracted epidemiological polarisation (32, 38). Agyei-Mensah and de-Graft Aikins described how Ghana had to deal simultaneously with persistent infectious diseases predominantly among poor people, increasing NCDs predominantly among wealthy people, and the emergence of the HIV/AIDS epidemic (32). Kahn et al. (38) examined trends in age-specific mortality in a rural South African population and reported a ‘counter transition’ of increasing mortality among children and young adults due to acute diarrhoea and malnutrition, ‘epidemiological polarisation’ from a greater mortality burden among disadvantaged groups, and a ‘protracted transition’ with a coexistence of HIV/AIDS and chronic NCDs in older adults (38, 39, 41). Peru also faces a double burden of persistent communicable diseases and NCDs, while dealing with injuries, re-emerging infections (TB, malaria), and HIV/AIDS. The country also suffers from inequalities in health and wealth within the country. This situation created an overlap of stages, broadening the gaps in health status among social classes and geographical regions (76, 77, 171).

### Determinants of ET

Omran postulated that ‘The shifts in health and disease patterns that characterize the epidemiologic transition are closely associated with the demographic and socioeconomic transition that constitute the modernization complex’. Demographic and socio-economic determinants, including sex, income level, education level, marital status, ethnicity, regional differences, as well as wider structural and environmental factors, can influence the transition process and lead to unequal health outcomes. In this review, we observed sex differentials in mortality over time (46, 49, 51, 73, 80, 81, 86, 94, 106, 118, 123, 132, 157); regional disparities in mortality (57, 60, 62, 72, 74, 76, 77, 85, 102, 146, 148, 172); social, economic, and mortality disparities across different ethnic groups (48, 49, 90, 98, 121, 125, 156); and climatic factors as determinants of mortality transition (115, 124, 153).

Although demographic and socio-economic transitions are significantly related to health improvement, some health problems still exist in some countries because the effects of socio-economic development and globalisation can be detrimental. In China, the economic reform that improved people's living environment (i.e. income and health care) had net effects on health. Conversely, economic reform in Russia did not produce any tangible economic and social benefits, and therefore health status was stagnant or deteriorating. In Russia, morbidity and mortality related to infectious disease increased after the reform, which could be explained by a relaxed or deteriorating public health network (152). Despite the improvement of socio-economic status in Chile during 1970–2000, Chile still experienced a persistent burden of tuberculosis, typhoid fever, and hepatitis, and at the same time, an increasing burden of HIV/AIDS (75). The association between unintentional injuries and economic development differed by age. In population groups aged 45–74 years, unintentional injuries were negatively associated with GNP per capita, reflecting lower rates of injuries in countries with high GDP. Among the group aged 75 years and above, the association was positive with a higher rate of unintentional injuries in countries with high GDP (137). The same pattern was observed for intentional injuries such as suicide (150).

### What deviations from the classic ET model have been observed, and do these reveal emerging patterns?

The process of mortality transition can be complex and dynamic, often influenced by demographic, socio-economic, technological, cultural, environmental, and biological drivers of change. We found numerous instances of deviations from the classic ET theory when examining specific geopolitical areas. A general assumption that current LMICs will experience the same transitional patterns as those countries already industrialised, following increased economic growth, is not proven. Some countries have encountered serious obstacles preventing them from completing certain stages of the transition, often tied to their history, economic development, or culture. Vallin and Mesle, referring to Eastern Europe and Africa, challenged the theory and proposed the idea that life expectancies are rapidly converging towards a maximum level (173). Having achieved relatively high life expectancies during the 1970s, a number of central European and former Soviet republics experienced periods of stagnation due to increasing cardiovascular mortality, violence, and alcoholism, with declines in life expectancy following the collapse of the USSR (97, 147, 174, 175). The combination of a period of highly centralised communist health planning, followed by a relatively disorderly transition towards free-market economies, was not a pattern that fitted into ET theory (175). In addition, the global economic crisis has also played important roles in this stagnation. Vallin and Mesle characterised the ‘cardiovascular revolution’ as a specific stage of ET when life expectancy increases and chronic diseases decline, which some countries in Eastern Europe have not yet achieved. Declining life expectancy in Eastern European countries is also closely linked to unusual distortions in the structure of age-specific mortality (173).

Many LMICs have started to close the gaps between themselves and more advanced countries in terms of economy, education, and health. While doing so, in the late 20th century, new infectious diseases such as HIV/AIDS and avian influenza showed remarkable abilities to spread as global epidemics. Recent outbreaks of avian influenza alerted the world to new susceptibilities to epidemics due to population growth, unplanned urbanisation, anti-microbial resistance, poverty, and lifestyle changes in communities. AIDS still persists as a major global health concern although medical technology has progressed in preventing new HIV infections and AIDS-related deaths. A global summary of the AIDS epidemic in 2011 showed a 15% increase in the number of people living with HIV but a 13% decrease in the number of people newly infected with HIV compared with 2001 (176). Although AIDS reporting mostly focuses on national trends, there are often large variations in HIV prevalence and epidemiological patterns within countries. The epidemic of HIV/AIDS appears to have stabilised in most countries, with sub-Saharan Africa remaining the most heavily affected region, accounting for 71% of newly infected HIV cases in 2010. The AIDS epidemic triggered a decrease, and in some cases a sharp drop, in the life expectancy levels in many African countries, which is bound to influence any interpretation of their progress in ET terms. The major demographic consequences of AIDS are something that Omran could not have possibly anticipated in 1971 but represent a salutary warning about the necessary complexity of any comprehensive and generalisable understanding of ET. The impact of wars and other forms of political violence, frequent in Africa, and perhaps most extreme in Somalia (177), introduces further uncertainty into understanding ET. In other cases, impacts may not be sufficiently population-wide to hugely influence ET. For example, HIV/AIDS in Thailand in the 1990s caused a 35% increase in adult male mortality but did not have a huge impact on life expectancy for the overall population in Thailand (101).

Declines in mortality in East Asian countries varied over space and time due to different political systems, different paces of economic growth, and geographical differences. East Asia underwent a significant mortality reduction in the second half of the 20th century. Japan began this rapid progress (91) and was followed by Taiwan, Hong Kong, Korea, and China (91, 158). Some African countries have advanced at a much slower pace than other countries with similar life expectancy levels. Many African countries have not yet reached the third stage of ET due to slow health care advances, and economic and political crises. Downward trends in mortality have been reversed in some areas as a consequence of the HIV/AIDS pandemic. In sub-Saharan Africa as a whole, as ET progresses problems tend to multiply, giving rise to the concept of the quadruple burden of disease (existing infectious diseases coupled with NCDs, injuries, and HIV/AIDS), which combine to disadvantage poor and marginalised groups (31, 39, 40, 42, 145, 161). We mapped some countries studied in this review to different sub-models which account for differences in timing and pace of ET in LMICs. The rapid sub-model related to countries such as Hong Kong and China, which experienced rapid industrialisation but remain in the third stage of ET (152, 155, 178–180). The intermediate sub-model refers to middle- or low-income countries, such as India, Vietnam, and Mexico, which still face overlapping infectious diseases and NCDs (17, 58, 60, 62, 63, 104, 181). This suggests that long-term shifts in patterns of mortality never entirely displace infectious diseases by degenerative and man-made disease and ageing, as Omran described in his theory. The slow sub-model refers to countries that are least prepared to handle the triple burden of disease, typically in Africa (38–41, 45).

We identified some new evidence of deviations from ET theory. According to the original theory, infectious diseases will gradually be eliminated as a major population health threat. However, there are examples to the contrary. In Nauru, previously high mortality from infectious diseases transitioned very rapidly into extremely high mortality from diabetes, circulatory disorders, and accidents over a short period, thus denying appreciable increases in life expectancy (132). Meanwhile, Mexico during 1922–1955 faced overlapping burdens of disease and an increasing trend of NCDs among the younger age groups, due to poverty and inability to afford healthcare (17). High mortality burdens from external causes, such as violence, in El Salvador did not follow cause of death patterns elsewhere which were predominated by infectious diseases at early stages of transition (182). Wolleswinkel-van den Bosch et al. showed in a cluster analysis in the Netherlands that the mortality decline in infectious diseases and the rise in non-infectious diseases had different time trends, in contrast with the classical interpretation of the ET that assumes uniform changes (113). Coste et al. evaluated the consistency of trends in causes of deaths in France in the short term. They found that there was a shift in mortality trends among adults during 1988–1999 that was marked by greater HIV infection, injury, and poisoning compared to mortality trends during 1968–1979. Their findings showed patterns of causes of deaths differing substantially from the classical patterns of ET (108). Mortality trends in the Tsimane communities in Bolivia showed decreased instances of deaths due to infectious diseases but increased instances of accidents and violence, particularly in the middle and late adulthood, which demonstrates a different pattern from the classical ET theory (105).

Another study in Hungary during 1971–2007 showed that the mortality patterns were different to what might be expected in the fourth stage of ET, in that female death rates increased due to ischaemic heart disease, while no evidence of emergent or re-emergent diseases were found and deaths related to external injuries declined (183). In Canada, the shifting pattern of causes of death did not entirely fit the theory. Lussier and Choiniere showed that decline in mortality related to cardiovascular disease was mostly concentrated in advanced ages, and the decline did not occur at the same pace for men and women (47). Fragmentation of the ET theory into distinctive stages delimited in time has proven to be inappropriate for Canada since the mid-20th century. The native Indian population in Canada has been shown to follow a different ET trajectory from the general population (52).

All these examples support existing criticisms that ET theory fails to describe the transition in some countries due to complexities of socio-economic, historical, political, and cultural factors that caused deviations from certain stages of the transition. Therefore, Omran's ET theory and its later developments cannot predict changes in mortality and cause-of-death patterns across all countries and time periods, as noted previously in some earlier reviews by Caselli et al. (10) and Gaylin and Kates (21). Despite these findings, we can conclude that relatively little research has concentrated on linking progress in ET to the original theory. The interest in the socio-economic determinants of ET has been greater than in the underlying disease shifts. Studies on mortality transitions in various societies still use theoretical perspectives to understand the process of population change by relating mortality patterns to demographic and socio-economic trends through the development of models (mechanisms of interaction that describe the patterns, determinants, and consequences of health and disease changes in diverse populations).

### Limitations of this study

One limitation of this paper is the potential of publication bias. We are aware that many of the published papers are papers that do not confirm to the ET theory, and hence perhaps had higher chances of being accepted for publication as the utility of the theory was debated. Papers with more ambiguous results may never be published. It is a challenge to use mortality to compare ETs across countries or regions, as many factors can influence the mortality transition. The use of age-standardised death rates might make the comparison across countries more compelling, though the question on the comparability of standard populations used remains valid.

## Conclusions

Despite many criticisms of Omran's theory, many researchers are still using the ET theory as the framework for their studies. In this review, we observe that the theory fits the transition patterns in some countries but needs further adjustments in other settings. With respect to broad categories, the original ET theory can, to some extent, continue to provide useful estimates of cause-specific mortality changes in countries where the transition started later than in industrialised countries and may contribute usefully to predictions of cause-specific mortality.

There are numerous examples where deviations from the classical ET theory have been observed in specific geopolitical settings since the theory was introduced four decades ago. Although the current state of knowledge and evidence on historical and contemporary cause-specific mortality changes may still be too sparse to formulate a new evidence-based theory that provides an integrated formulation of the underlying processes of cause-specific mortality change, the need to update the ET theory is clear.

A comprehensive theory must start from the complexity of reality, even though it may not always be possible to measure all necessary parameters; a phenomenological basis will not be adequate. A new evidence-based formulation in terms of patterns of changes in causes of death, and disruptions in health due to emerging risks, is needed, which focuses on the underlying mechanisms and cause-specific mortality changes that result, rather than the current crude description of a decline in infectious diseases and a rise in non-infectious diseases with little reference to underlying determinants. Since comprehensive data are not available on a 100% basis around the world, judicious use will need to be made of the best available sources of evidence, such as those coming, for example, from the long-term surveillance of defined populations by the INDEPTH Network in otherwise uncounted regions (184). At the same time, optimal use needs to be made of data archives which document ET processes already experienced. The underlying processes of mortality change need to be described more specifically with reference to causes of death, speed of mortality changes, factors that cause disruption to health, and the ways in which populations adapt to these disruptions. Comparative analyses using standardised methods in various population groups must provide information for elaborating and refining models of transition, as is needed to handle at least some of the many problems associated with changes in population inequalities.

**Main findings**

- Experiences of epidemiological transition have varied between countries in terms of timing, pace and underlying mechanisms.
- Elements of epidemiological transition have been described in many countries, but observed transitions have not always followed the pathways described in Omran's original theory.
- The emphasis of epidemiological transition research has changed over the past four decades, with an increasing tendency to study wide-ranging aspects of the determinants of mortality, including risk factors, lifestyle changes, socio-economics and macro factors such as climate change.

**Key messages for action**

- Though it does not completely describe transition patterns observed in all settings, the original ET theory can continue to provide a useful framework for describing cause-specific mortality changes and may contribute usefully to predictions of cause-specific mortality, particularly in low- and middle-income settings.
- There is need for common consensus on a vital minimum dataset, such as a complete civil registration and documentation of major risk factors and health care coverage in low and middle-income countries to better document changing health patterns and support national health information systems. In the meantime, the lack of comprehensive population health and demographic data necessitates judicious use of the available data sources.
- Revisions to epidemiological transition theory are needed and should be based on the growing empirical evidence base in a wide range of settings.

## References

[1] Kirk D (1996). Demographic transition theory. Popul Stud.

[2] Thompson WS (1929). Population. Am J Sociol.

[3] Landry A (1987). The demographic revolution. Pol Popul Rev.

[4] Notestein F, Schultz TW (1945). Population – the long view. Food for the world.

[5] Omran AR (1971). The epidemiologic transition. A theory of the epidemiology of population change. Milbank Mem Fund Q.

[6] Omran AR (1983). The epidemiologic transition theory. A preliminary update. J Trop Pediatr.

[7] Omran AR (1998). The epidemiologic transition theory revisited thirty years later. World Health Stat Q.

[8] Olshansky SJ, Ault AB (1986). The fourth stage of the epidemiologic transition: the age of delayed degenerative diseases. Milbank Q.

[9] Rogers RG, Hackenberg R (1987). Extending epidemiologic transition theory: a new stage. Soc Biol.

[10] Caselli G, Meslé F, Vallin J (2002). Epidemiologic transition theory exceptions. Genus.

[11] Armelagos GJ, Brown PJ, Turner B (2005). Evolutionary, historical and political economic perspectives on health and disease. Soc Sci Med.

[12] Gage TB (2005). Are modern environments really bad for us?: revisiting the demographic and epidemiologic transitions. Am J Phys Anthropol.

[13] Weisz G, Olszynko-Gryn J (2010). The theory of epidemiologic transition: the origins of a citation classic. J Hist Med Allied Sci.

[14] Kunitz SJ (1990). The value of particularism in the study of the cultural, social and behavioral determinants of mortality. What we know about health transition. The cultural, social, and behavioral determinants of health.

[15] Ruzicka L, Kane P (1990). Health transition: the course of morbidity and mortality. Determinants Health J.

[16] Fetter B, Coello-Ramirez P, Rogers J, Nelson M (1997). Forum: the epidemiolocal transition. Forum Health Trans Rev.

[17] Carolina Martinez S, Gustavo Leal F (2003). Epidemiological transition: model or illusion? A look at the problem of health in Mexico. Soc Sci Med.

[18] Frenk J, Bobadilla JL, Stern C, Frejka T, Lozano R (1991). Elements for a theory of the health transition. Health Trans Rev.

[19] Smallman-Raynor M, Phillips D (1999). Late stages of epidemiological transition: health status in the developed world. Health Place.

[20] Mackenbach JP (1994). The epidemiologic transition theory. J Epidemiol Commun Health.

[21] Gaylin DS, Kates J (1997). Refocusing the lens: epidemiologic transition theory, mortality differentials, and the AIDS pandemic. Soc Sci Med.

[22] Caldwell JC, Caldwell P (1991). What have we learnt about the cultural, social and behavioural determinants of health? From selected readings to the first Health Transition Workshop. Health Trans Rev.

[23] Caldwell JC (2001). Population health in transition. Bull World Health Organ.

[24] McKeown T (1989). The road to health. World Health Forum.

[25] Preston S (1975). The changing relation between mortality and socioeconomic development. Pop Stud.

[26] Caldwell JC (1993). Health transition: the cultural, social and behavioural determinants of health in the Third World. Soc Sci Med.

[27] Caldwell JC, Caldwell P (1999). Epidemiologic transitions. Kaohsiung J Med Sci.

[28] Mathers CD, Fat DM, Inoue M, Rao C, Lopes AD (2005). Counting the dead and what they died from: an assessment of the global status of cause of death data. Bull World Health Organ.

[29] Alter GC, Carmichael AG (1997). Reflections on the classification of causes of death. Contin Chang.

[30] Berhane Y, Wall S, Fantahun M, Emmelin A, Mekonnen W, Hogberg U (2008). A rural Ethiopian population undergoing epidemiological transition over a generation: Butajira from 1987 to 2004. Scand J Public Health.

[31] Misganaw A, Mariam DH, Araya T (2012). The double mortality burden among adults in Addis Ababa, Ethiopia, 2006–2009. Prevent Chronic Dis.

[32] Agyei-Mensah S, de-Graft Aikins A (2010). Epidemiological transition and the double burden of disease in Accra, Ghana. J Urban Health.

[33] Chihana M, Floyd S, Molesworth A, Crampin AC, Kayuni N, Price A (2012). Adult mortality and probable cause of death in rural northern Malawi in the era of HIV treatment. Trop Med Int Health.

[34] Bah SM (1998). Assessing the contribution of age-sex differentials in causes of death due to infectious and parasitic diseases to the trends in age-sex differentials in life expectancy in Mauritius. Soc Biol.

[35] Abdesslam B (2012). Evolution of rural-urban health gaps in Morocco: 1992–2011. BMC Res Notes.

[36] Bovet P (1995). The epidemiologic transition to chronic diseases in developing countries: cardiovascular mortality, morbidity, and risk factors in Seychelles (Indian Ocean). Investigators of the Seychelles Heart Study. Soz Praventivmed.

[37] Stringhini S, Sinon F, Didon J, Gedeon J, Paccaud F, Bovet P (2012). Declining stroke and myocardial infarction mortality between 1989 and 2010 in a country of the african region. Stroke.

[38] Kahn K, Garenne ML, Collinson MA, Tollman SM (2007). Mortality trends in a new South Africa: hard to make a fresh start. Scand J Public Health Suppl.

[39] Tollman SM, Kahn K, Sartorius B, Collinson MA, Clark SJ, Garenne ML (2008). Implications of mortality transition for primary health care in rural South Africa: a population-based surveillance study. Lancet.

[40] Sartorius B, Kahn K, Collinson MA, Vounatsou P, Tollman SM (2011). Survived infancy but still vulnerable: spatial-temporal trends and risk factors for child mortality in the Agincourt rural sub-district, South Africa, 1992–2007. Geospat Health.

[41] Sartorius BK, Kahn K, Vounatsou P, Collinson MA, Tollman SM (2010). Young and vulnerable: spatial-temporal trends and risk factors for infant mortality in rural South Africa (Agincourt), 1992–2007. BMC Public Health.

[42] Bradshaw D, Groenewald P, Laubscher R, Nannan N, Nojilana B, Norman R (2003). Initial burden of disease estimates for South Africa, 2000. S Afr Med J.

[43] Garenne M, Kahn K, Collinson M, Gomez-Olive X, Tollman S (2013). Protective effect of pregnancy in rural South Africa: questioning the concept of “indirect cause” of maternal death. PLoS One.

[44] Kahn K, Tollman SM, Garenne M, Gear JS (1999). Who dies from what? Determining cause of death in South Africa's rural north-east. Trop Med Int Health.

[45] Garenne M, Gakusi E (2006). Health transitions in sub-Saharan Africa: overview of mortality trends in children under 5 years old (1950–2000). Bull World Health Organ.

[46] Narh-Bana SA, Chirwa TF, Mwanyangala MA, Nathan R (2012). Adult deaths and the future: a cause-specific analysis of adult deaths from a longitudinal study in rural Tanzania 2003–2007. Trop Med Int Health.

[47] Lussier MH, Bourbeau R, Choiniere R (2008). Does the recent evolution of Canadian mortality agree with the epidemiologic transition theory?. Demogr Res.

[48] Trovato F (1988). Mortality differentials in Canada, 1951–1971: French, British, and Indians. Cult Med Psychiatry.

[49] Trovato F (2000). Canadian Indian mortality during the 1980s. Soc Biol.

[50] Young TK (1988). Are subarctic Indians undergoing the epidemiologic transition?. Soc Sci Med.

[51] Chen J, Millar WJ (2000). Are recent cohorts healthier than their predecessors?. Health Rep.

[52] Marrett LD, Chaudhry M (2003). Cancer incidence and mortality in Ontario First Nations, 1968–1991 (Canada). Cancer Causes Control.

[53] Diaz-Briquets S (1981). Determinants of mortality transition in developing countries before and after the Second World War: some evidence from Cuba. Popul Stud.

[54] Rodriguez-Ojea A, Jimenez S, Berdasco A, Esquivel M (2002). The nutrition transition in Cuba in the nineties: an overview. Public Health Nutr.

[55] Rosero-Bixby L (1991). Socioeconomic development, health interventions and mortality decline in Costa Rica. Scand J Soc Med Suppl.

[56] Perry H, Berggren W, Berggren G, Dowell D, Menager H, Bottex E (2007). Long-term reductions in mortality among children under age 5 in rural Haiti: effects of a comprehensive health system in an impoverished setting. Am J Public Health.

[57] McCaw-Binns A, Alexander SF, Lindo JL, Escoffery C, Spence K, Lewis-Bell K (2007). Epidemiologic transition in maternal mortality and morbidity: new challenges for Jamaica. Int J Gynaecol Obstet.

[58] Malina RM, Pena Reyes ME, Little BB (2008). Epidemiologic transition in an isolated indigenous community in the Valley of Oaxaca, Mexico. Am J Phys Anthropol.

[59] Pick JB, Butler EW (1998). Demographic, social, and economic effects on Mexican causes of death in 1990. Soc Biol.

[60] Stevens G, Dias RH, Thomas KJ, Rivera JA, Carvalho N, Barquera S (2008). Characterizing the epidemiological transition in Mexico: national and subnational burden of diseases, injuries, and risk factors. PLoS Med.

[61] Little BB, Malina RM, Reyes ME (2008). Natural selection and demographic transition in a Zapotec-speaking genetic isolate in the Valley of Oaxaca, southern Mexico. Ann Hum Biol.

[62] Barquera S, Tovar-Guzman V, Campos-Nonato I, Gonzalez-Villalpando C, Rivera-Dommarco J (2003). Geography of diabetes mellitus mortality in Mexico: an epidemiologic transition analysis. Arch Med Res.

[63] Rivera JA, Barquera S, Campirano F, Campos I, Safdie M, Tovar V (2002). Epidemiological and nutritional transition in Mexico: rapid increase of non-communicable chronic diseases and obesity. Public Health Nutr.

[64] Gulliford MC (1996). Epidemiological transition in Trinidad and Tobago, West Indies 1953–1992. Int J Epidemiol.

[65] Mungrue K (2012). The changing face of death in Trinidad and Tobago, before and after independence. West Indian Med J.

[66] Condran GA, Cheney RA (1982). Mortality trends in Philadelphia: age- and cause-specific death rates, 1870–1930. Demography.

[67] Fliess KH (1991). Mortality transition among the Wends of Serbin, Texas, 1854–1884: changes in pattern of death from parochial records. Soc Biol.

[68] Potter LB (1991). Socioeconomic determinants of white and black males’ life expectancy differentials, 1980. Demography.

[69] Mansur Ade P, Lopes AI, Favarato D, Avakian SD, Cesar LA, Ramires JA (2009). Epidemiologic transition in mortality rate from circulatory diseases in Brazil. Arq Bras Cardiol.

[70] Barros FC, Victora CG, Vaughan JP, Tomasi E, Horta BL, Cesar JA (2001). The epidemiological transition in maternal and child health in a Brazilian city, 1982–93: a comparison of two population-based cohorts. Paediatr Perinat Epidemiol.

[71] Chaimowicz F (2001). Age transition of tuberculosis incidence and mortality in Brazil. Rev Saude Publica.

[72] Sastry N (2004). Trends in socioeconomic inequalities in mortality in developing countries: the case of child survival in Sao Paulo, Brazil. Demography.

[73] Guimaraes RM, Muzi CD (2012). Trend of mortality rates for gastric cancer in Brazil and regions in the period of 30 years (1980–2009). Arq Gastroenterol.

[74] Moura EC, Pacheco-Santos LM, Peters LR, Serruya SJ, Guimaraes R (2012). Research on chronic noncommunicable diseases in Brazil: meeting the challenges of epidemiologic transition. Rev Panam Salud Publica.

[75] Albala C, Vio F (1995). Epidemiological transition in Latin America: the case of Chile. Public Health.

[76] Huicho L, Trelles M, Gonzales F, Mendoza W, Miranda J (2009). Mortality profiles in a country facing epidemiological transition: an analysis of registered data. BMC Public Health.

[77] Huynen MM, Vollebregt L, Martens P, Benavides BM (2005). The epidemiologic transition in Peru. Rev Panam Salud Publica.

[78] Pineros M, Hernandez G, Bray F (2004). Increasing mortality rates of common malignancies in Colombia: an emerging problem. Cancer.

[79] Yang G, Kong L, Zhao W, Wan X, Zhai Y, Chen LC (2008). Emergence of chronic non-communicable diseases in China. Lancet.

[80] Yang G, Wang Y, Zeng Y, Gao GF, Liang X, Zhou M (2013). Rapid health transition in China, 1990–2010: findings from the Global Burden of Disease Study 2010. Lancet.

[81] Jiang G, Wang D, Li W, Pan Y, Zheng W, Zhang H (2012). Coronary heart disease mortality in China: age, gender, and urban-rural gaps during epidemiological transition. Rev Panam Salud Publica.

[82] Chan MF, Van IK, Ng WI (2010). Factors contributing to neonatal mortality rates in Macao: evidence from 1957–2006 data. Nurs Health Sci.

[83] Gao J, Qian J, Tang S, Eriksson BO, Blas E (2002). Health equity in transition from planned to market economy in China. Health Policy Plan.

[84] Schooling CM, Lau EW, Tin KY, Leung GM (2010). Social disparities and cause-specific mortality during economic development. Soc Sci Med.

[85] Joshi R, Cardona M, Iyengar S, Sukumar A, Raju CR, Raju KR (2006). Chronic diseases now a leading cause of death in rural India – mortality data from the Andhra Pradesh Rural Health Initiative. Int J Epidemiol.

[86] Kumar R, Kumar D, Jagnoor J, Aggarwal AK, Lakshmi PV (2012). Epidemiological transition in a rural community of northern India: 18-year mortality surveillance using verbal autopsy. J Epidemiol Community Health.

[87] Palanivel C, Yadav K, Gupta V, Rai SK, Misra P, Krishnan A (2013). Causes of death in rural adult population of North India (2002–2007), using verbal autopsy tool. Indian J Public Health.

[88] Arokiasamy P, Gautam A (2008). Neonatal mortality in the empowered action group states of India: trends and determinants. J Biosoc Sci.

[89] Pradhan J, Arokiasamy P (2010). Socio-economic inequalities in child survival in India: a decomposition analysis. Health Policy.

[90] Keinan-Boker L, Baron-Epel O, Fishler Y, Liphshitz I, Barchana M, Dichtiar R (2013). Breast cancer trends in Israeli Jewish and Arab women, 1996–2007. Eur J Cancer Prev.

[91] Kuroda T (1978). The demographic transition in Japan. Soc Sci Med.

[92] Yong V, Saito Y (2012). Are there education differentials in disability and mortality transitions and active life expectancy among Japanese older adults? Findings from a 10-year prospective cohort study. J Gerontol B Psychol Sci Soc Sci.

[93] Suh I (2001). Cardiovascular mortality in Korea: a country experiencing epidemiologic transition. Acta Cardiol.

[94] Lee ES (1986). Epidemiologic transition in Korea: a new perspective in population and development studies. Health Policy.

[95] Lee ES (1986). Mortality transition in Korea: its implications for health policy and education. Health Policy.

[96] Abu-Rmeileh NM, Husseini A, Abu-Arqoub O, Hamad M, Giacaman R (2008). Mortality patterns in the West Bank, Palestinian Territories, 1999–2003. Prev Chronic Dis.

[97] Vishnevsky A, Shkolnikov V, Vassin S (1991). Epidemiological transition in the USSR as mirrored by regional differences. Genus.

[98] Niti M, Ng TP (2001). Temporal trends and ethnic variations in amenable mortality in Singapore 1965–1994: the impact of health care in transition. Int J Epidemiol.

[99] Meegama SA (1986). The mortality transition in Sri Lanka. Determinants of Mortality Change and Differentials in Developing Countries: The Five-Country Case Study Project. Population Studies, No. 94; ST/ESA/SER.A/94.

[100] Bundhamcharoen K, Odton P, Phulkerd S, Tangcharoensathien V (2011). Burden of disease in Thailand: changes in health gap between 1999 and 2004. BMC Public Health.

[101] Hill K, Vapattanawong P, Prasartkul P, Porapakkham Y, Lim SS, Lopez AD (2007). Epidemiologic transition interrupted: a reassessment of mortality trends in Thailand, 1980–2000. Int J Epidemiol.

[102] Akgun S, Rao C, Yardim N, Basara BB, Aydin O, Mollahaliloglu S (2007). Estimating mortality and causes of death in Turkey: methods, results and policy implications. Eur J Public Health.

[103] Huong DL, Minh HV, Byass P (2003). Applying verbal autopsy to determine cause of death in rural Vietnam. Scand J Public Health Suppl.

[104] Hoa NP, Rao C, Hoy DG, Hinh ND, Chuc NT, Ngo DA (2012). Mortality measures from sample-based surveillance: evidence of the epidemiological transition in Viet Nam. Bull World Health Organ.

[105] Gurven M, Kaplan H, Supa AZ (2007). Mortality experience of Tsimane Amerindians of Bolivia: regional variation and temporal trends. Am J Hum Biol.

[106] Georgieva L, Powles J, Genchev G, Salchev P, Poptodorov G (2002). Bulgarian population in transitional period. Croat Med J.

[107] Koupilova I, McKee M, Holcik J (1998). Neonatal mortality in the Czech Republic during the transition. Health Policy.

[108] Coste J, Bernardin E, Jougla E (2006). Patterns of mortality and their changes in France (1968–99): insights into the structure of diseases leading to death and epidemiological transition in an industrialised country. J Epidemiol Community Health.

[109] Balogh S, Papp R, Jozan P, Csaszar A (2010). Continued improvement of cardiovascular mortality in Hungary – impact of increased cardio-metabolic prescriptions. BMC Public Health.

[110] Kalediene R, Petrauskiene J (2005). Inequalities in mortality by education and socio-economic transition in Lithuania: equal opportunities?. Public Health.

[111] Kalediene R, Petrauskiene J, Starkuviene S (2007). Inequalities in mortality by marital status during socio-economic transition in Lithuania. Public Health.

[112] Van Poppel F, Schellekens J, Liefbroer AC (2002). Religious differentials in infant and child mortality in Holland, 1855–1912. Popul Stud.

[113] Wolleswinkel-van den Bosch JH, Looman CW, Van Poppel FW, Mackenbach JP (1997). Cause-specific mortality trends in The Netherlands, 1875–1992: a formal analysis of the epidemiologic transition. Int J Epidemiol.

[114] Alfonso Sanchez MA, Mendietat VP, Pena JA, Calderon R (2002). Demographic and health patterns in a rural community from the Basque area in Spain (1800–1990). J Biosoc Sci.

[115] Munoz-Tuduri M, Garcia-Moro C, Walker PL (2006). Time series analysis of the epidemiological transition in Minorca, 1634–1997. Hum Biol.

[116] Alfonso-Sanchez MA, Calderon R, Pena JA (2004). Opportunity for natural selection in a Basque population and its secular trend: evolutionary implications of epidemic mortality. Hum Biol.

[117] Alfonso-Sanchez MA, Pena JA, Calderon R (2003). Time trends and determinants of completed family size in a rural community from the Basque area of Spain (1800–1969). J Biosoc Sci.

[118] Moraes SA, Suzuki CS, Freitas IC, Costa Junior ML (2009). Mortality rates due to diseases of the circulatory system (DCS) in Ribeirao Preto – SP, from 1980 to 2004. Arq Bras Cardiol.

[119] Moreno LA, Sarria A, Popkin BM (2002). The nutrition transition in Spain: a European Mediterranean country. Eur J Clin Nutr.

[120] Skold P, Axelsson P (2008). The northern population development; colonization and mortality in Swedish Sapmi, 1776–1895. Int J Circumpolar Health.

[121] Skold P, Axelsson P, Karlsson L, Smith L (2011). Infant mortality of Sami and settlers in Northern Sweden: the era of colonization 1750–1900. Glob Health Action.

[122] Demetrius L (1989). The demographic evolution of human populations: the role of selection and environmental factors. Demography.

[123] Hemstrom O (1999). Explaining differential rates of mortality decline for Swedish men and women: a time-series analysis, 1945–1992. Soc Sci Med.

[124] Carson C, Hajat S, Armstrong B, Wilkinson P (2006). Declining vulnerability to temperature-related mortality in London over the 20th century. Am J Epidemiol.

[125] Wild S, Fischbacher C, Brock A, Griffiths C, Bhopal R (2007). Mortality from all causes and circulatory disease by country of birth in England and Wales 2001–2003. J Public Health.

[126] Burnley IH (1998). Inequalities in the transition of ischaemic heart disease mortality in New South Wales, Australia, 1969–1994. Soc Sci Med.

[127] Burnley IH, Rintoul D (2002). Inequalities in the transition of cerebrovascular disease mortality in New South Wales, Australia 1969–1996. Soc Sci Med.

[128] Carter K, Cornelius M, Taylor R, Ali SS, Rao C, Lopez AD (2011). Mortality trends in Fiji. Aust N Z J Public Health.

[129] Vigneron E (1989). The epidemiological transition in an overseas territory: disease mapping in French Polynesia. Soc Sci Med.

[130] Vigneron E (1993). Epidemiological transition and geographical discontinuities: the case of cardiovascular mortality in French Polynesia. Soc Sci Med.

[131] Carter K, Soakai TS, Taylor R, Gadabu I, Rao C, Thoma K (2011). Mortality trends and the epidemiological transition in Nauru. Asia Pac J Public Health.

[132] Schooneveldt M, Songer T, Zimmet P, Thoma K (1988). Changing mortality patterns in Nauruans: an example of epidemiological transition. J Epidemiol Community Health.

[133] Davis P (1984). Health patterns in New Zealand: class, ethnicity and the impact of economic development. Soc Sci Med.

[134] Sandiford P (2009). Getting back the missing men of Aotearoa: declining gender inequality in NZ life expectancy. J Prim Health Care.

[135] Ahmed N, Andersson R (2000). Unintentional injury mortality and socio-economic development among 15–44-year-olds: in a health transition perspective. Public Health.

[136] Moniruzzaman S, Andersson R (2004). Relationship between economic development and suicide mortality: a global cross-sectional analysis in an epidemiological transition perspective. Public Health.

[137] Moniruzzaman S, Andersson R (2005). Relationship between economic development and risk of injuries in older adults and the elderly. A global analysis of unintentional injury mortality in an epidemiologic transition perspective. Eur J Public Health.

[138] Plitponkarnpim A, Andersson R, Jansson B, Svanstrom L (1999). Unintentional injury mortality in children: a priority for middle income countries in the advanced stage of epidemiological transition. Inj Prev.

[139] Heuveline P, Guillot M, Gwatkin DR (2002). The uneven tides of the health transition. Soc Sci Med.

[140] Serow WJ, Cowart ME, Camezon J (1998). Epidemiologic transition theory and aging: Hispanic populations of North America and the Caribbean. J Health Hum Serv Adm.

[141] Shah A (2010). The possible evidence for an epidemiological transition hypothesis for elderly suicides. Int Psychogeriatr.

[142] Viner RM, Coffey C, Mathers C, Bloem P, Costello A, Santelli J (2011). 50-year mortality trends in children and young people: a study of 50 low-income, middle-income, and high-income countries. Lancet.

[143] Murray CJ, Lopez AD (1997). Mortality by cause for eight regions of the world: Global Burden of Disease Study. Lancet.

[144] Murray CJ, Lopez AD (1997). Global mortality, disability, and the contribution of risk factors: Global Burden of Disease Study. Lancet.

[145] Lopez AD, Mathers CD (2006). Measuring the global burden of disease and epidemiological transitions: 2002–2030. Ann Trop Med Parasitol.

[146] Dhillon PK, Jeemon P, Arora NK, Mathur P, Maskey M, Sukirna RD (2012). Status of epidemiology in the WHO South-East Asia region: burden of disease, determinants of health and epidemiological research, workforce and training capacity. Int J Epidemiol.

[147] Hofmarcher MM (1998). Is public health between East and West? Analysis of wealth, health and mortality in Austria, Central and Eastern European Countries and Croatia relative to the European Union. Croat Med J.

[148] Pérez-Farinós N, López-Abente G, Pastor-Barriuso R (2006). Time trend and age-period-cohort effect on kidney cancer mortality in Europe, 1981–2000. BMC Public Health.

[149] Lozano R, Naghavi M, Foreman K, Lim S, Shibuya K, Aboyans V (2012). Global and regional mortality from 235 causes of death for 20 age groups in 1990 and 2010: a systematic analysis for the Global Burden of Disease Study 2010. Lancet.

[150] Moniruzzaman S, Andersson R (2008). Cross-national injury mortality differentials by income level: the possible role of age and ageing. Public Health.

[151] Kaasik T, Andersson R, Horte LG (1998). The effects of political and economic transitions on health and safety in Estonia: an Estonian-Swedish comparative study. Soc Sci Med.

[152] Liu Y, Rao K, Fei J (1998). Economic transition and health transition: comparing China and Russia. Health Policy.

[153] Fellman J, Eriksson AW (2001). Regional, temporal, and seasonal variations in births and deaths: the effects of famines. Soc Biol.

[154] Phillips DR (1991). Problems and potential of researching epidemiological transition: examples from Southeast Asia. Soc Sci Med.

[155] Zhao D, Liu J, Wang W, Zeng Z, Cheng J, Liu J (2008). Epidemiological transition of stroke in China: twenty-one-year observational study from the Sino-MONICA-Beijing Project. Stroke.

[156] Trovato F (2001). Aboriginal mortality in Canada, the United States and New Zealand. J Biosoc Sci.

[157] Weden MM, Brown RA (2006). Historical and life course timing of the male mortality disadvantage in Europe: epidemiologic transitions, evolution, and behavior. Soc Biol.

[158] Zhao Z, Kinfu Y (2005). Mortality transition in East Asia. Asian Popul Stud.

[159] Mackenbach JP (2013). Convergence and divergence of life expectancy in Europe: a centennial view. Eur J Epidemiol.

[160] Adjuik M, Smith T, Clark S, Todd J, Garrib A, Kinfu Y (2006). Cause-specific mortality rates in sub-Saharan Africa and Bangladesh. Bull World Health Organ.

[161] Fottrell E, Kahn K, Ng N, Sartorius B, Huong DL, Van Minh H (2010). Mortality measurement in transition: proof of principle for standardised multi-country comparisons. Trop Med Int Health.

[162] Pendleton BF, Yang SO (1985). Socioeconomic and health effects on mortality declines in developing countries. Soc Sci Med.

[163] Taylor R, Bampton D, Lopez AD (2005). Contemporary patterns of Pacific Island mortality. Int J Epidemiol.

[164] Winegarden CR, Murray JE (2004). Effects of early health-insurance programs on European mortality and fertility trends. Soc Sci Med.

[165] Lewis ME (2002). Impact of industrialization: comparative study of child health in four sites from medieval and postmedieval England (A.D. 850–1859). Am J Phys Anthropol.

[166] Murray CJ, Bobadilla JL, Bobadilla JL, Costello CA, Mitchell F (1997). Epidemiological transitions in the former socialist economies: divergent patterns of mortality and causes of death. Premature death in the new independent states.

[167] Kintner HJ (1999). Recording the epidemiologic transition in Germany, 1816–1934. J Hist Med Allied Sci.

[168] Spijker JJ, Camara AD, Blanes A (2012). The health transition and biological living standards: adult height and mortality in 20th-century Spain. Econ Hum Biol.

[169] Bah SM (1995). Quantitative approaches to detect the fourth stage of the epidemiologic transition. Soc Biol.

[170] Verastegui E, Mohar A (2010). Colorectal cancer in Mexico: should a middle income country invest in screening or in treatment?. Eur J Health Econ.

[171] Fraser B (2006). Peru's epidemiological transition. Lancet.

[172] Gupte MD, Ramachandran V, Mutatkar RK (2001). Epidemiological profile of India: historical and contemporary perspectives. J Biosci.

[173] Vallin J, Mesle F (2004). Convergences and divergences in mortality. A new approach to health transition. Demogr Res.

[174] Shkolnikov V, McKee M, Leon D, Chenet L (1999). Why is the death rate from lung cancer falling in the Russian Federation?. Eur J Epidemiol.

[175] Veenema TG (2000). Health systems and maternal and child survival in Central Asian Republics. J Nurs Scholarsh.

[176] World Health Organization (2011). Global HIV/AIDS response: epidemic update and health sector progress towards universal access.

[177] Guha-Sapir D, Ratnayake R (2009). Consequences of ongoing civil conflict in Somalia: evidence for public health responses. PLoS Med.

[178] Gu D, Dupre ME, Warner DF, Zeng Y (2009). Changing health status and health expectancies among older adults in China: gender differences from 1992 to 2002. Soc Sci Med.

[179] Cook IG, Dummer TJ (2004). Changing health in China: re-evaluating the epidemiological transition model. Health Policy.

[180] Wu P, Cowling BJ, Schooling CM, Wong IO, Johnston JM, Leung CC (2008). Age-period-cohort analysis of tuberculosis notifications in Hong Kong from 1961 to 2005. Thorax.

[181] Guerra-Godinez JC, Larrosa-Haro A, Coello-Ramirez P, Tostado HR, Rivera-Chavez E, Castillo de Leon YA (2003). Changing trends in prevalence, morbidity, and lethality in persistent diarrhea of infancy during the last decade in Mexico. Arch Med Res.

[182] Avilles L (2001). Epidemiology as discourse: the politics of development institutions in the Epidemiological Profile of El Salvador. J Epidemiol Community Health.

[183] Kovacs K, Hablicsek L (2010). Cause specific mortality trends in Hungary in the light of the theory of the epidemiological transition.

[184] Sankoh O, Byass P (2012). The INDEPTH Network: filling vital gaps in global epidemiology. Int J Epidemiol.

